# Genetic and Phenotypic Comparison of Facultative Methylotrophy between *Methylobacterium extorquens* Strains PA1 and AM1

**DOI:** 10.1371/journal.pone.0107887

**Published:** 2014-09-18

**Authors:** Dipti D. Nayak, Christopher J. Marx

**Affiliations:** 1 Organismic and Evolutionary Biology, Harvard University, Cambridge, Massachusetts, United States of America; 2 Faculty of Arts and Sciences Center for Systems Biology, Harvard University, Cambridge, Massachusetts, United States of America; 3 Department of Biological Sciences, University of Idaho, Moscow, Idaho, United States of America; 4 Institute for Bioinformatics and Evolutionary Studies, University of Idaho, Moscow, Idaho, United States of America; Texas A&M, United States of America

## Abstract

*Methylobacterium extorquens* AM1, a strain serendipitously isolated half a century ago, has become the best-characterized model system for the study of aerobic methylotrophy (the ability to grow on reduced single-carbon compounds). However, with 5 replicons and 174 insertion sequence (IS) elements in the genome as well as a long history of domestication in the laboratory, genetic and genomic analysis of *M. extorquens* AM1 face several challenges. On the contrary, a recently isolated strain - *M. extorquens* PA1- is closely related to *M. extorquens* AM1 (100% 16S rRNA identity) and contains a streamlined genome with a single replicon and only 20 IS elements. With the exception of the methylamine dehydrogenase encoding gene cluster (*mau)*, genes known to be involved in methylotrophy are well conserved between *M. extorquens* AM1 and *M. extorquens* PA1. In this paper we report four primary findings regarding methylotrophy in PA1. First, with a few notable exceptions, the repertoire of methylotrophy genes between PA1 and AM1 is extremely similar. Second, PA1 grows faster with higher yields compared to AM1 on C_1_ and multi-C substrates in minimal media, but AM1 grows faster in rich medium. Third, deletion mutants in PA1 throughout methylotrophy modules have the same C_1_ growth phenotypes observed in AM1. Finally, the precision of our growth assays revealed several unexpected growth phenotypes for various knockout mutants that serve as leads for future work in understanding their basis and generality across *Methylobacterium* strains.

## Introduction

Methylotrophy is the ability of microorganisms to grow on reduced single-carbon (C_1_) compounds such as CH_4_ (methane) or CH_3_OH (methanol) as a sole carbon and energy source [1]–[4]. *Methylobacterium extorquens* AM1 [1] is a facultative methylotroph that belongs to the *Rhizobiales* family of the Alpha-proteobacteria. Since *M. extorquens* AM1 is genetically tractable [5]–[12], has fast, roughly comparable growth rates on C_1_ compounds (t_D_∼3–4 h on methanol and methylamine) and multi-carbon compounds (t_D_∼3 h on succinate) [13], [14], it has emerged as the model system for the study of aerobic methylotrophy [15], [16]. There are three specific aspects of the genome architecture and physiology of AM1 that pose challenges [17]–[24]. First, the AM1 genome has five replicons of different sizes [17]. One of the replicons in the AM1 genome is a 1.3 Mb megaplasmid that contains many insertion sequence (IS) elements; recombination events mediated by IS elements often lead to large, beneficial deletions [18]. Hence, experiments designed to study a variety of questions have and will commonly result in this particular change of large benefit [18]. Second, the 174 intact or partial IS elements across 39 IS families present in the AM1 genome [16], [19] lead to genomic plasticity. In fact, a large number of IS mediated recombination events have often been observed during genetic manipulations and evolution experiments with AM1 [20]–[23]. Such high rates of IS insertion/recombination in AM1 leads to spurious recombination events across the genome during reverse genetic manipulations (Nayak, Carroll, and Marx; unpublished) and skews the mutational spectrum during experimental evolution [18]. Third, the current strain of AM1 has been domesticated in laboratory conditions since the late 1950s [1]–[3] and the growth characteristics of the ‘modern’ strain have changed [24]. A notable difference is that the ‘modern’ strain [9] grows ∼25% worse than an archival version under a wide variety of conditions. These results indicate that aspects of physiology uncovered in the ‘modern’ AM1 may be hard to extrapolate to other environmentally relevant methylotrophs.

Of late, an increasing number of studies have been conducted with several members of the *M. extorquens* species and genome sequence data is now available for six strains [17], [25]. Despite 16S rRNA sequence similarity, these strains vary in terms of their metabolic breadth, genetic tractability, ecological niche and genomic composition [25]. We considered whether one of these six sequenced strains might overcome the challenges posed by AM1 and finally narrowed in on *M. extorquens* PA1 (hereafter PA1) as it has the most streamlined genome, with a single 5.47 Mb chromosome, and contains only 20 intact IS elements. These features can make the design and implementation of genetic screens more efficient, and prevent beneficial elimination of extra-chromosomal elements or IS-mediated events from dominating the spectrum of beneficial mutations. Recent isolation of PA1 from the leaves of *Arabidopsis thaliana* [26], and immediate cryopreservation obviate concerns associated with domestication of the ‘modern’ AM1 strain and provide a clear link to a known ecological niche.

**Figure 1:**
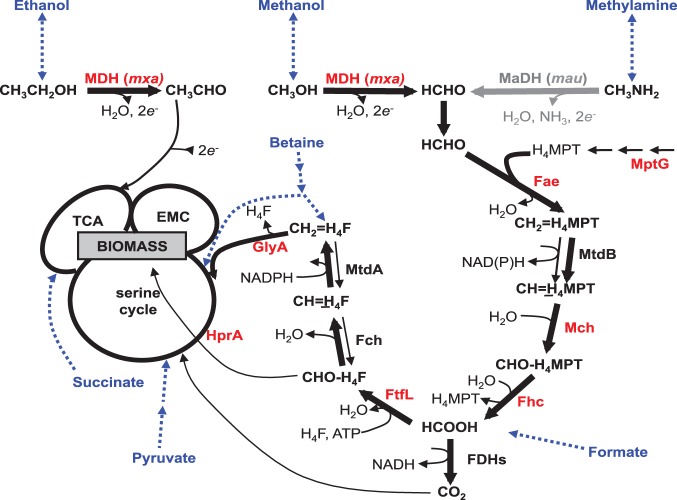
The methylotrophy specific metabolic network in in *M. extorquens* AM1. All genes, except for the *mau* cluster (gray), are present and >95% identical in *M. extorquens* PA1. *M. extorquens* AM1 and *M. extorquens* PA1 were grown on various C_1_ and multi-C substrates (blue) for this study. Genes highlighted in red were deleted in *M. extorquens* PA1 to uncover that the metabolic network involved in methylotrophy in *M. extorquens* PA1 is identical to *M. extorquens* AM1. TCA: Tricarboxylic acid Cycle and EMC: Ethyl-malonyl CoA Pathway.

There are some clear advantages of the genome composition and culturing history of PA1 over AM1. In order to ascertain how well the decades of characterization of methylotrophy in AM1 will apply directly to PA1, we identified the shared repertoire of methylotrophy genes and performed a broad genetic analysis of the role of various methylotrophy modules in PA1 (Figure 1). In AM1, reduced C_1_ compounds such as methanol or methylamine are oxidized by dedicated periplasmic dehydrogenases to generate formaldehyde. Once in the cytoplasm, formaldehyde is oxidized to formate via a tetrahydromethanopterin (H_4_MPT) dependent pathway [27], [28]. Formate is then either oxidized to CO_2_ via a panel of formate dehydrogenases [29]), or is assimilated into biomass via a tetrahydrofolate (H_4_F) dependent pathway [30]–[33]. The C_1_ unit from methylene-H_4_F (an intermediate of the H_4_F pathway) along with an equal amount of CO_2_ [34] is assimilated into biomass via the serine cycle [15] and the ethylmalonyl-CoA pathway [34].

The genetic and phenotypic analysis in this work demonstrated that the vast body of knowledge pertaining to methylotrophy in *M. extorquens* AM1 is largely transferable to *M. extorquens* PA1: an alternate model system for the study of aerobic methylotrophy in the future. Additionally, our quantitative physiological analysis has also unveiled novel phenotypes for methylotrophy-specific genes that generate leads to uncover poorly understood aspects of regulation in future work.

## Materials and Methods

### Bacterial Strains and Growth Conditions

The Δ*cel* mutant of the pink-pigmented ‘wildtype’ stock of AM1 (CM2720) and the Δ*cel* mutant of the pink-pigmented ‘wildtype’ stock of PA1 (CM2730) used for growth comparisons are described elsewhere [13]. Standard growth conditions utilized a modified version of Hypho minimal medium consisting of: 100 mL phosphate salts solution (25.3 g of K_2_HPO_4_ plus 22.5 g Na_2_HPO_4_ in 1L deionized water), 100 mL sulfate salts solution (5 g of (NH_4_)_2_SO_4_ and 2 g of MgSO_4_ • 7 H_2_O in 1 L deionized water), 799 mL of deionized water, and 1 mL of trace metal solution [35]. All components were autoclaved separately before mixing under sterile conditions. Filter-sterilized carbon sources were added just prior to inoculation in liquid minimal media with a final concentration of either15 mM methanol, 3.5 mM sodium succinate, 15 mM methylamine hydrochloride, 7.5 mM ethanol, 5 mM sodium pyruvate, 15 mM glycine betaine, 7.5 mM methanol and 1.75 mM succinate, or Difco nutrient broth. Difco nutrient broth (Becton, Dickson and Company, Franklin Lakes, NJ) was prepared according to the manufacturer’s guidelines.

### Growth Rate Measurements

All *M. extorquens* strains were acclimated, grown in 48-well microtiter plates (CoStar-3548) in an incubation tower (Liconic USA LTX44 with custom fabricated cassettes) shaking at 650 rpm, in a room that was constantly maintained at 30°C and 80% humidity [36], containing Hypho medium with the appropriate carbon source to a volume of 640 µL. All growth regimes consisted of three cycles consisting of inoculation, acclimation, and growth measurement. All strains were stored in vials at −80°C in 10% DMSO; growth was initiated by transferring 10 µL freezer stock into 10 mL of Hypho medium with 3.5 mM succinate. Upon reaching stationary phase (∼2 days), cultures were transferred 1∶64 into fresh medium with the carbon source to be tested, allowed to reach saturation in this acclimation phase, and diluted 1∶64 again into fresh medium for the measured (experimental) growth. The increase in OD_600_ for strains grown in 48-well microtiter plates was measured using an automated, robotic culturing and monitoring system [13], [36]. A series of robotic instruments (including a shovel, a transfer station, and a twister arm), all controlled by an open-source control program, Clarity [36], were used to move the 48-well plates from the incubation tower (Liconic USA LTX44 with custom fabricated cassettes) to a Perkin-Elmer Victor2 plate reader for optical density (OD_600_) measurements. The dynamics and specific growth rate of cultures were calculated from the log-linear growth phase using an open source, custom-designed growth analysis software called CurveFitter available at http://www.evolvedmicrobe.com/CurveFitter/(Figure S3). Growth rates reported for each strain and condition are the mean plus SEM calculated from triplicate biological replicates, unless otherwise noted.

### Generation of Mutant Strains

*M. extorquens* PA1 deletion mutants lacking the *mxa* operon, *fae*, *mptG*, *ftfL*, *glyA*, or *hprA* (Figure 1, Table 1) were generated on the genetic background of CM2730 using the allelic exchange vector pCM433 [9]. The double deletion mutants lacking *mptG* and *mch* or *fhcBACD* were generated on the genetic background of CM3803 (Δ*mptG* in CM2730) using the allelic exchange vector pCM433 [9]. A region upstream and downstream of each of these genes or operons of ∼0.5 kb was amplified using PCR. The forward primer for the upstream flank was designed to have a 30 bp long sequence at the 5′ end homologous to the sequence upstream of the *Not*I cut site in pCM433. The reverse primer for the upstream flank was designed to have a 30 bp sequence at the 5′ end homologous to the first 30 bp of the downstream flank. The reverse primer for the downstream flank was designed to have a 30 bp long sequence at the 5′ end homologous to the sequence downstream of the *Not*I cut site in pCM433. The PCR products representing the upstream and downstream flank were ligated on the pCM433 vector cut with *Not*I using the Gibson assembly protocol described elsewhere [37]. Cloning the upstream and downstream flanks for *fae*, *ftfL, glyA, mptG,* the *mxa* operon, *fhc*BACD, *mch,* and *hprA* in pCM433 resulted in pDN50, pDN56, pDN66, pDN68, pDN94, pDN108, pDN109, and pDN125, respectively (Table S9). Mutant strains of *M. extorquens* PA1 were made by introducing the appropriate donor constructs through conjugation by a tri-parental mating between the competent *E. coli* NEB 10β (New England Biolabs, Ipswich, MA) containing the donor construct, an *E. coli* strain containing the conjugative plasmid pRK2073 [38], and PA1 as described elsewhere [9]. All mutant strains were confirmed by diagnostic PCR analysis and validated by Sanger sequencing the mutant locus. All strains and plasmids used and generated for this study are listed in Table 1.

**Table 1:** *M. extorquens* strains and plasmids used in this study.

Strain orplasmid	Description	Reference
CM2720	Δ*cel M. extorquens* AM1	[13]
CM2730	Δ*cel M. extorquens* PA1	[13]
CM3753	Δ*fae* in CM2730	This study
CM3773	Δ*ftfL* in CM2730	This study
CM3799	Δ*glyA* in CM2730	This study
CM3803	Δ*mptG* in CM2730	This study
CM3849	Δ*mxa* operon in CM2730	This study
CM3889	Δ*fhcBACD*, Δ*mptG* in CM2730	This study
CM3891	Δ*mch*, Δ*mptG* in CM2730	This study
CM4122	Δ*hprA* in CM2730	This study
pCM433	Allelic exchange vector (Amp^R^, Chl^R^, Tet^R^, Suc^S^)	[9]
pDN50	pCM433 with Δ*fae* upstream anddownsteam flanks	This study
pDN56	pCM433 with Δ*ftfL*upstream anddownsteam flanks	This study
pDN66	pCM433 with Δ*glyA* upstream and downsteam flanks	This study
pDN68	pCM433 with Δ*mptG* operon upstreamand downsteam flanks	This study
pDN94	pCM433 with Δ*mxa* operon upstream anddownsteam flanks	This study
pDN108	pCM433 with Δ*fhcBACD* upstream anddownsteam flanks	This study
pDN109	pCM433 with Δ*mch* upstream anddownsteam flanks	This study
pDN125	pCM433 with Δ*hprA* upstream anddownsteam flanks	This study
pRK2073	Conjugative helper plasmid (Str^R^)	[38]

## Results and Discussion

### Comparison of methylotrophy genes in PA1 versus AM1

As a first step to compare methylotrophy in PA1 and AM1, we considered the content, similarity and organization of genes in each genome. Apart from 100% identity at the16S rRNA locus [25], the two strains also share 95.9% ITS (Internal Transcribed Spacer) 1 sequence identity, each has five *rrn* operons, and their GC contents are quite similar (68.2% versus 68.5%). Of the identified 5333 coding sequences in the PA1 genome, 4260 are shared with AM1 (amino acid identity >30%). Of the 90 genes known to be involved in methylotrophy, 62 have >99% identity and the remaining 28 have at least 95% identity at the amino acid level between AM1 and PA1 (Table S1). This repertoire includes the genes involved in methanol oxidation, the H_4_MPT- and H_4_F-dependent C_1_- transfer pathways, the four formate dehydrogenases, and genes of the serine cycle (Figure 1). The arrangement of genes is extremely similar between the chromosomes of AM1 and PA1 (Figure S1). There is one major difference: the cluster of genes encoding methylamine dehydrogenase (*mau)* is missing in PA1. The *mau* cluster in AM1 and *M. extorquens* CM4 is flanked by IS elements, and is also missing in another sequenced strain, *M. extorquens* DM4. These data further support the hypothesis that the *mau* cluster was acquired by horizontal gene transfer [17], [18].

### Phenotypic comparison of growth on C_1_ and multi-C substrates for PA1 versus AM1

Even though methylotrophy-specific genes are shared and extremely similar between PA1 and AM1, it does not necessarily translate into quantitatively similar growth phenotypes on C_1_- or multi-C substrates. In order to rigorously compare the growth capabilities of the two strains, we took advantage of the recent development of an automated, robotic platform for high-throughput, quantitative measurements of *M. extorquens* growth [13], [36]. No significant difference in cell shape, cell size and biomass/OD_600_ ratio was observed between PA1 and AM1 (Table S2) hence maximum OD_600_ during growth was used as a proxy for yield. Additionally, for all phenotypic analyses we used strains of PA1 and AM1 that lacked the *cel* locus for cellulose biosynthesis. The Δ*cel* manipulation prevents ‘clumping’ of cells in 48-well plates, thereby made growth measurements more accurate and consistent [13].

**Figure 2:**
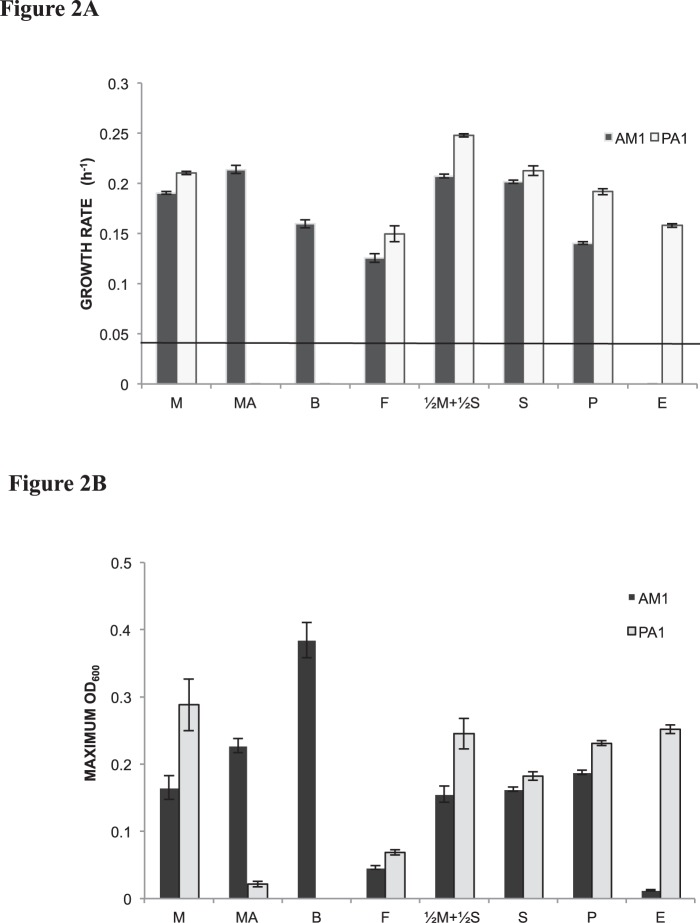
Quantitative comparison of growth rates and maximum OD_600_ values. **A)** Growth rates for the Δ*cel* ‘wild-type’ strain of AM1 (filled) versus the Δ*cel* ‘wild-type’ strain of PA1 (open) on C_1_ substrates (M, 15 mM methanol; MA, 15 mM methylamine; F, 15 mM formate), the joint C_1_ and multi-C substrate betaine (B, 15 mM), multi-carbon substrates (S, 3.5 mM succinate; P, 5 mM pyruvate; E, 7.5 mM ethanol) and a combination of C_1_ and multi-carbon substrates (½M+½S, 1.75 mM succinate and 7.5 mM methanol). Error bars represent the 95% C.I. of the average of three biological replicates. The line indicates the approximate detection limit of our automated growth rate measurement device of 0.04 hr^−1^. Growth rates for PA1 on MA or B, and for AM1 on E were below this detection limit. **B)** Maximum OD_600_ for the Δ*cel* ‘wild-type’ strain of AM1 (filled) versus the Δ*cel* ‘wild-type’ strain of PA1 (open) on C_1_ substrates (M, 15 mM methanol; MA, 15 mM methylamine; F, 15 mM formate), the joint C_1_ and multi-C substrate betaine (B, 15 mM), multi-carbon substrates (S, 3.5 mM succinate; P, 5 mM pyruvate; E, 7.5 mM ethanol) and a combination of C_1_ and multi-carbon substrates (½M+½S, 1.75 mM succinate and 7.5 mM methanol). Error bars represent the 95% C.I. (confidence interval) of the average of three biological replicates.

With a few notable exceptions, PA1 grew faster, with significantly higher yields, than AM1 on C_1_ and multi-C substrates (Figure 2a and 2b). PA1 grew 10–15% faster, with 50–75% higher yield compared to AM1 on methanol as well as formate. On ethanol, a doubling time of 4.39 h was observed for PA1, but AM1 barely grew (Figure 2b); we were unable to reliably estimate the doubling time for AM1 since it was below the detection limit (t_D_ ∼17.5 h) of our growth measurement platform. At a genomic level, such a striking difference in ethanol growth rates might be due to specific genes downstream of primary oxidation, such as an aldehyde dehydrogeanse (Mext_1295), present in PA1 but absent in AM1. On multi-C organic acids, PA1 grew faster than AM1 by 5–25% with 12–25% higher yield.

In contrast to the results above, AM1 grew faster than PA1 on two C_1_ substrates: methylamine and betaine (*N, N, N-* tri-methyl glycine). A small but significant increase in OD_600_ (Figure 2b) indicated that PA1 can grow on methylamine, but the growth rate was extremely slow and below the detection limit of our growth measurement platform. This observation is consistent with the slow methylamine growth known for other organisms solely dependent upon the *N*-methylglutamate pathway for methylamine utilization [39], [40]. Specific proteins involved in betaine transport and utilization have not been discovered in AM1, so we can speculate that these genes may be missing or insufficiently active in PA1. Growth in rich media i.e. Nutrient Broth did not have the typical log-linear dynamics that displays a consistent, quantifiable growth rate (Figure S2) because: a) Nutrient Broth is a composite of many different growth substrates and b) Nutrient Broth is not buffered so the pH of the media changes drastically over the course of growth. However, since AM1 reached stationary phase much before PA1, it was evident that AM1 grew significantly faster than PA1 (Figure S2). As previously hypothesized [24], faster growth of AM1 on Nutrient Broth may stem from ‘laboratory adaptation’ since AM1 was stored on nutrient agar slants [41] in the refrigerator for prolonged periods of time prior to cryopreservation. These conditions could have led to cryptic nutrient cycling of a wide variety of compounds, perhaps even lysed cell material, by surviving lineages [24].

### Genetic characterization of methylotrophy in PA1

In order to probe the architecture of the metabolic network involved in methylotrophy in PA1 and ascertain how similar it is to that described for AM1 (Figure 1), we deleted from the Δ*cel* PA1 strain (referred to as WT from here on) key genes involved each methylotrophy-specific module and examined the resulting growth phenotypes on C_1_, multi-C, and a combination of C_1_ and multi-C substrates (Table S3–S8).

#### Methanol oxidation

**Figure 3:**
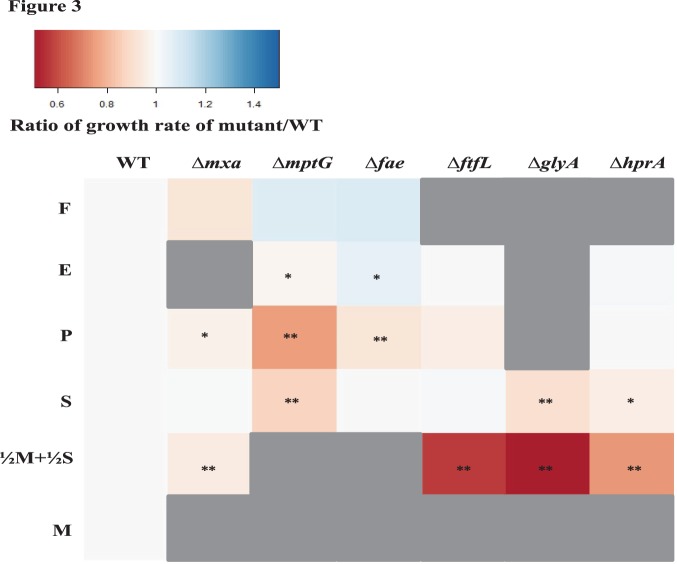
Heat map depicting the ratio of growth rate of knockout mutants of PA1 relative to the growth rate of the Δ*cel* wild-type strain on C_1_ or multi-C substrates (same concentrations as in **Figure 2**). Undetectable growth is indicated by grey. A significant difference (determined by comparing the mean growth rate of three biological replicates using the t-test) in growth rate with a p-value <0.05 is indicated by a * and a p-value <0.01 is indicated by **.

**Figure 4:**
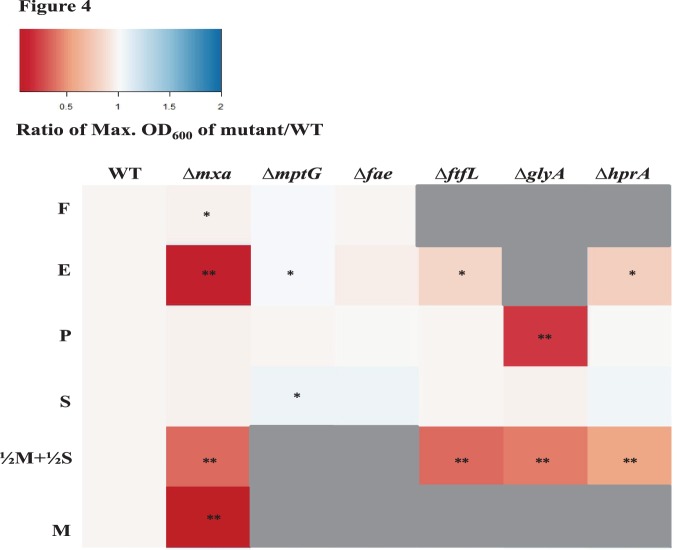
Heat map depicting the ratio of maximum OD_600_ of knockout mutants of PA1 relative to the maximum OD_600_ of the Δ*cel* wild-type strain on several C_1_ or multi-C substrates (same concentrations as in **Figure 2**). Maximum OD_600_ values below 0.01 are indicated by grey. A significant difference in maximum OD_600_ (determined by comparing the mean growth rate of three biological replicates using the t-test) with a p-value <0.05 is indicated by a * and a p-value <0.01 is indicated by **.

There are 15 methanol oxidation genes in AM1, as well as in PA1. In AM1, 14 of these genes are co-transcribed [42], including those encoding the large and small subunit (MxaFI) of methanol dehydrogenase [43] and ancillary proteins involved in transport, assembly and electron transfer [15]. Deleting the *mxa* operon in PA1 led a to a drastic growth defect on methanol (Figure 3) demonstrating that MDH is, the primary enzyme involved in methanol oxidation. The Δ*mxa* mutant of PA1 had a severe growth defect on ethanol as well (Figure 3 and 4, Table S6–S8). This observation supported a hypothesis, based on *in vitro* studies [44], that MDH in *M. extorquens* strains can catalyze the oxidation of ethanol *in vivo*. Slow growth (as indicated by an increase in yield in Figure 4, Table S6–S8) for the Δ*mxa* mutant on methanol or ethanol indicated that alternate, physiologically relevant alcohol dehydrogenase(s) for each of these substrates exist in the PA1 genome.

#### Formaldehyde oxidation

Genetic and biochemical analyses have determined that the tetrahydromethanopterin (H_4_MPT) dependent pathway is the sole route for the oxidation of formaldehyde to formate in AM1 [7], [28], [31] (Figure 1). In order to determine if the H_4_MPT dependent pathway is required for formaldehyde oxidation in PA1, we individually deleted two key genes of this pathway: *mptG* (encoding ribofuranosylaminobenzene 5′-phosphate synthase that catalyzes the first step of the H_4_MPT biosynthesis pathway [45]) and *fae* (encoding the formaldehyde-activating enzyme that catalyzes the condensation of formaldehyde and H_4_MPT [46]). Deleting either *mptG* or *fae* in PA1 abolished growth on methanol (Figure 3). As observed in AM1 [28], we suspect PA1 mutants lacking theH_4_MPT pathway were sensitive to methanol because of the toxic effects of formaldehyde buildup [28]. Additionally, we noted that these two mutants grew slower (without any yield defect) on multi-C compounds; the Δ*mptG* mutant had a more severe growth-rate defect than the Δ*fae* mutant (Figure 3, Table S3–S5). For example, on pyruvate, the Δ*mptG* mutant grew 25% slower (p<0.001; Student’s two-sided t-test with n = 3) and the Δ*fae* mutant grew 7% slower (p<0.01) than WT. These results are consistent with, and build upon, previous work in AM1 that qualitatively demonstrated that the Δ*mptG* mutant has a growth defect on succinate [28]. Furthermore, ethanol growth was not abolished in formaldehyde oxidation mutants, suggesting that the overlap between methanol and ethanol growth includes just primary oxidation but not any further oxidation steps (Table S3–S8).

As observed in AM1 [28], deletions in the genes encoding the final two enzymes of the H_4_MPT pathway, *mch* and *fhc* [47]–[49], could only be generated in strains with a lesion in *mptG* [45]. This result is consistent with the hypothesis [28] that a late block in the H_4_MPT mediated formaldehyde oxidation pathway leads to the accumulation of either methylene- or methenyl-H_4_MPT, which may be either be directly toxic and/or lead to a regulatory response halting growth.

#### Formate assimilation

In order to determine the role of the H_4_F mediated C_1_ transfer pathway during growth on C_1_ substrates, we deleted *ftfL* (encoding formate-H_4_F ligase) [30] in PA1. (Figure 3, Figure 4, Table S3–S8). The Δ*ftfL* mutant in PA1 could not grow on methanol or formate, most likely because of a lesion in the first dedicated step toward assimilation of C_1_ compounds [31], [32] (Figure 3). However, the Δ*ftfL* PA1 mutant, unlike the Δ*ftfL* AM1 mutant [50], did not have a significant growth rate or yield advantage on multi-C compounds (Table S3–S5).

#### Serine cycle

Carbon from C_1_ substrates is converted to various components of biomass through the serine cycle in AM1 [4], [15]. To determine whether the serine cycle plays a key role during C_1_ assimilation in PA1, we deleted *glyA* (serine hydroxymethyltransferase), and *hprA* (hydroxypyruvate reductase) (Figure 1) [15]. As in AM1, neither mutant could grow on any C_1_ substrates (Figure 3). While the Δ*hprA* strain had WT-like growth characteristics on multi-C compounds, the Δ*glyA* mutant exhibited several unexpected phenotypes: a complete inability to grow on ethanol, extremely slow growth on pyruvate and a 10% decrease (p<0.01) in growth rate on succinate compared to WT (Figure 3, Figure 4, Table S3–S8). These results suggest that alternative pathway(s) used to generate C_1_-H_4_F intermediates during multi-C growth, such as glycine cleavage [51], only partially rescue growth in the Δ*glyA* strain. Future work will be required to understand why the magnitude of growth defects on ethanol, pyruvate, and succinate varies for the Δ*glyA* mutant.

### Growth on the combination of C_1_- and multi-C substrates

**Figure 5:**
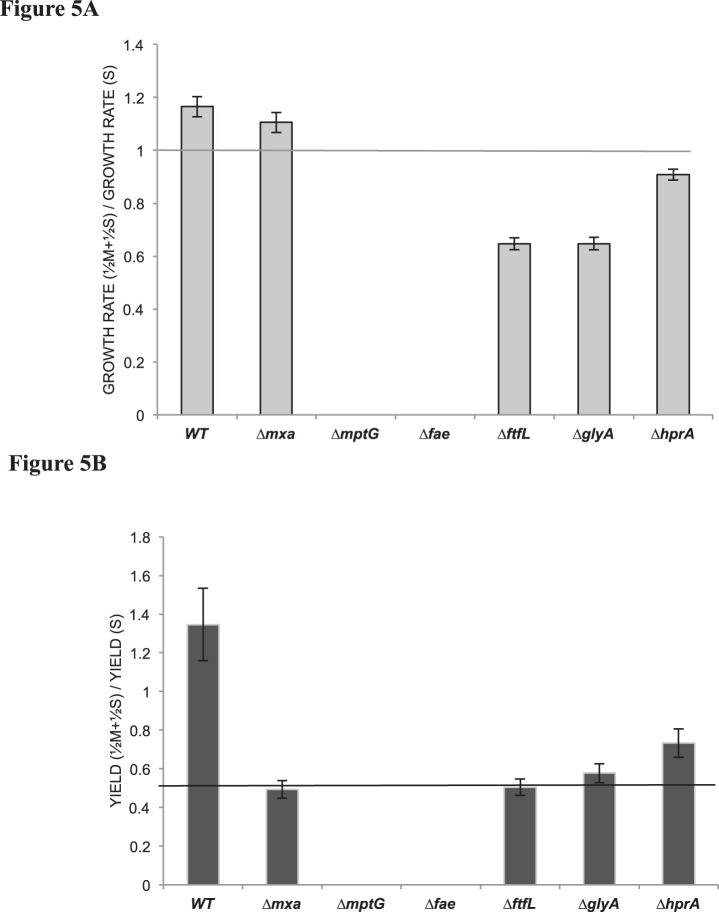
Comparison of growth rates and maximum OD600 values on methanol with succinate versus on succinate alone. **A)** Ratio of growth rates, of the Δ*cel* ‘wildtype’ strain of PA1 and various knockout mutants, on a combination of ½M+½S (7.5 mM methanol+1.75 mM succinate) versus S (3.5 mM succinate). The dotted line depicts the expected ratio for growth rate, if no methanol was oxidized in a combination of M+S. **B)** Ratio of yield (measured as the maximum OD_600_ value during growth), for the Δ*cel* ‘wildtype’ strain of PA1 and various knockout mutants, on M+S versus S. The dotted line depicts expected ratio for yields, if no methanol was assimilated in a combination of M+S. For all data error bars represent the 95% C.I. of the average ratio of three biological replicates grown in each condition.

The major goal of this study was to compare the metabolic network involved in C_1_- and multi-C metabolism in PA1 to that established for AM1. In addition, however, we also uncovered a number of unexpected growth phenotypes, especially on the combination of C_1_- and multi-C substrates. In contrast to a previous study [52], which showed that AM1 grows on a combination of succinate and methanol at the same rate as succinate or methanol, PA1 grew 16% faster, with a 35% increase in yield, on a combination of methanol and succinate, compared to succinate alone (Figure 5a and 5b). The growth pattern suggested that cells utilized methanol and succinate simultaneously; as was previously observed in AM1 as well [52]. Since growth on succinate is energy-limited and growth on methanol is reducing-power limited [52], [53], it is likely that growth and yield on methanol and succinate is greater because the combination compensates for limitations posed by each substrate in isolation.

Based on the growth characteristics of mutants in methylotrophy specific modules, we hypothesized likely phenotypes during growth on the combination of C_1_ and multi-C substrates. Since the Δ*mxa* mutant is incapable of methanol growth, we anticipated that its growth rate on a combination of methanol and succinate would be the same as that on succinate, but with 50% lower yield since the combination contains half the concentration of succinate and methanol. While the observed yield on the combination matched the expected value, the Δ*mxa* mutant grew 11% faster (p<0.001) on the combination than on succinate alone (Figure 5a and 5b). One possibility is that methanol is either being sensed or oxidized by the XoxFI system; an MxaFI homolog suggested to play a regulatory role in *M. extorquens* [54], [55] and works as a lanthanide dependent methanol dehydrogenase in the acidiphilic methanotroph *Methylacidiphilum fumariolicum* SolV [56]. We hypothesized that the C_1_ assimilation mutants (i.e., Δ*ftfL,* Δ*glyA,* and *ΔhprA* mutants) might be able to grow faster on a combination of methanol and succinate because these mutants are capable of completely oxidizing methanol to generate reducing power. Furthermore, the ability to generate additional reducing power from methanol might also result in a proportional increase in the yield. Although yields on the combination of methanol and succinate were significantly elevated for the Δ*glyA* and Δ*hprA* strains, growth rate was compromised relative to succinate growth (Δ*ftfL* and Δ*glyA* by 35% and Δ*hprA* by 9%; Figure 4a). This partial methanol sensitivity, though not as severe as seen for H_4_MPT mutants, may be due to build-up of (potentially toxic) C_1_ intermediates and/or a regulatory mismatch between C_1_ dissimilation and succinate assimilation. Future work will be required to understand the physiological basis for these effects, and to determine whether they hold for other strains of *Methylobacterium*.

## Conclusions

Since extremely closely-related strains sometimes occupy distinct niches [57] and have distinct metabolic capabilities, we felt it was critical to compare the growth of PA1 and AM1 on a range of substrates in order to establish just how much confidence one should have in transferring knowledge gained from the long-studied AM1 to the newer option of PA1. The stark difference in growth rate and yield on certain substrates, such as betaine, methylamine, and ethanol, between the two strains might reflect adaptation/s to unique ecological niches by each of these strains prior to isolation, such as via recent gain or loss of certain metabolic genes or differential regulation [58].

Our genetic analysis of methylotrophy in PA1 establishes that the roles of the various methylotrophy specific modules during C_1_ growth are the same as described in AM1. In addition, owing to the quantitative nature of our analyses, several knockout mutants also revealed unexpected phenotypes on multi-C compounds as well as a combination of C_1_ and multi-C compounds. These phenotypes point towards yet-undiscovered aspects of metabolism in these facultative methylotrophs in terms of regulation that allows cells to switch between C_1_ metabolism and multi-C growth, as well as to establish balanced growth on multiple substrates simultaneously.

## Supporting Information

Figure S1 **A line plot of strand conservation (in purple) and strand inversion (in blue) between the chromosome of PA1 and the main chromosome of AM1 (bottom).**
(PDF)

Figure S2 **Growth of three biological replicates of the Δ** ***cel*** **‘wild-type’ strain of PA1 (gray) and the Δ** ***cel*** **‘wild-type’ strain of AM1 (black) in nutrient broth.** The inset shows the semi-log plot of the growth curves to emphasize the deceleration in growth.
(PDF)

Figure S3 **Growth curves of three replicates of the Δ** ***cel*** **strain of PA1 (WT) (in green), the Δ** ***mxa*** **mutant of WT (in red), and the Δ** ***glyA*** **mutant of WT (in blue) on a combination of 7.5 mM methanol and 1.75 mM succinate as seen in the open-source growth curve fitter software, Curve Fitter.**
(PDF)

Table S1 **A list of methylotrophy-specific genes shared between** ***M. extorquens*** **strains AM1 and PA1.** Green: genes involved in methanol oxidation, pink: genes involved in the H_4_MPT dependent formaldehyde oxidation pathway; purple: genes involved in the H_4_F-dependent formate reduction pathway; orange: genes encoding each of the four formate dehydrogenases; and gray: C_1_ assimilation genes.
(PDF)

Table S2 **Mean FSC (Forward Scatter) and SSC (Side Scatter) of 50,000 cells of** ***M. extorquens*** **AM1 and PA1 (grown in 3.5 mM succinate) using a flow cytometer.** FSC is an estimate for the relative size of the cell and SSC is an estimate for the granularity or the biomass/OD_600_ ratio of the cell. Values are reported as mean±95% confidence interval of the mean for three independent flow cytometer runs with 50,000 cells each.
(PDF)

Table S3 **Mean growth rates (in h^−1^) and the standard error of the mean growth rates on C_1_ substrates M (15 mM methanol), MA (15 mM methylamine), F (15 mM formate) for AM1 and PA1 (both lacking the** ***cel*** **locus), as well as the mutants strains of Δ** ***cel*** **PA1.**
(PDF)

Table S4 **Mean growth rates (in h^−1^) and the standard srror of the mean growth rates on multi-C substrates S (3.5 mM succinate), P (5 mM pyruvate), E (7.5 mM ethanol) for AM1 and PA1 (both lacking the** ***cel*** **locus), as well as the mutants strains of Δ** ***cel*** **PA1.**
(PDF)

Table S5 **Mean growth rates (in h^−1^) and the standard error of the mean growth rates on a joint C_1_ and multi-C substrate B (15 mM betaine), or a combination of C_1_ and multi-C substrates ½M+½S (7.5 mM methanol and 1.75 mM succinate) for AM1 and PA1 (both lacking the** ***cel*** **locus), as well as the mutants strains of Δ** ***cel*** **PA1.**
(PDF)

Table S6 **Max OD_600_ and the standard error of the max OD_600_ on C_1_ substrates M (15 mM methanol), MA (15 mM methylamine), F (15 mM formate) for AM1 and PA1 (both lacking the** ***cel*** **locus), as well as the mutants strains of Δ** ***cel*** **PA1.**
(PDF)

Table S7 **Mean max OD_600_ and the standard error of the max OD_600_ on multi-C substrates S (3.5 mM succinate), P (5 mM pyruvate), E (7.5 mM ethanol) for AM1 and PA1 (both lacking the** ***cel*** **locus), as well as the mutants strains of Δ** ***cel*** **PA1.**
(PDF)

Table S8 **Mean max OD_600_ and the standard error of the max OD_600_ on a joint C_1_ and multi-C substrate B (15 mM betaine), or a combination of C_1_ and multi-C substrates ½M+½S (7.5 mM methanol and 1.75 mM succinate) for AM1 and PA1 (both lacking the** ***cel*** **locus), as well as the mutants strains of Δ** ***cel*** **PA1.**
(PDF)

Table S9 **List of primers used for deleting methylotrophy-specific genes in** ***M. extorquens*** **PA1.**
(PDF)
