# Supplementary material for: Genetic and Phenotypic Comparison of Facultative Methylotrophy between Methylobacterium extorquens Strains PA1 and AM1
Source: PLoS One. 2014 Sep 18;9(9):e107887. doi: 10.1371/journal.pone.0107887 (PMC4169470; doi:10.1371/journal.pone.0107887)
Supplement: Figure S1 — A line plot of strand conservation (in purple) and strand inversion (in blue) between the chromosome of PA1 and the main chromosome of AM1 (bottom). (PDF) [file pone.0107887.s001.pdf]

Figure S1: A line plot of strand conservation (in purple) and strand inversion (in blue) between the chromosome of PA1 and the main chromosome of AM1 (bottom) generated using the 'Conserved Synteny line plot' with synton size ≥ 3 genes using the Microscope genomics platform (http://www.cns.fr/agc/microscope/home/index.php).

***M. extorquens* PA1**

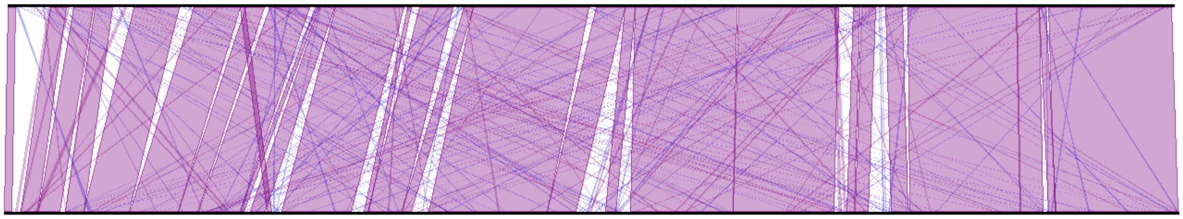

***M. extorquens* AM1**
