# Supplementary material for: Genetic and Phenotypic Comparison of Facultative Methylotrophy between Methylobacterium extorquens Strains PA1 and AM1
Source: PLoS One. 2014 Sep 18;9(9):e107887. doi: 10.1371/journal.pone.0107887 (PMC4169470; doi:10.1371/journal.pone.0107887)

Figure S2: Growth of three biological replicates of the Δ*cel* 'wild-type' strain of PA1 (gray) and the Δ*cel* 'wild-type' strain of AM1 (black) in nutrient broth. The inset shows the semi-log plot of the growth curves to emphasize the deceleration in growth.

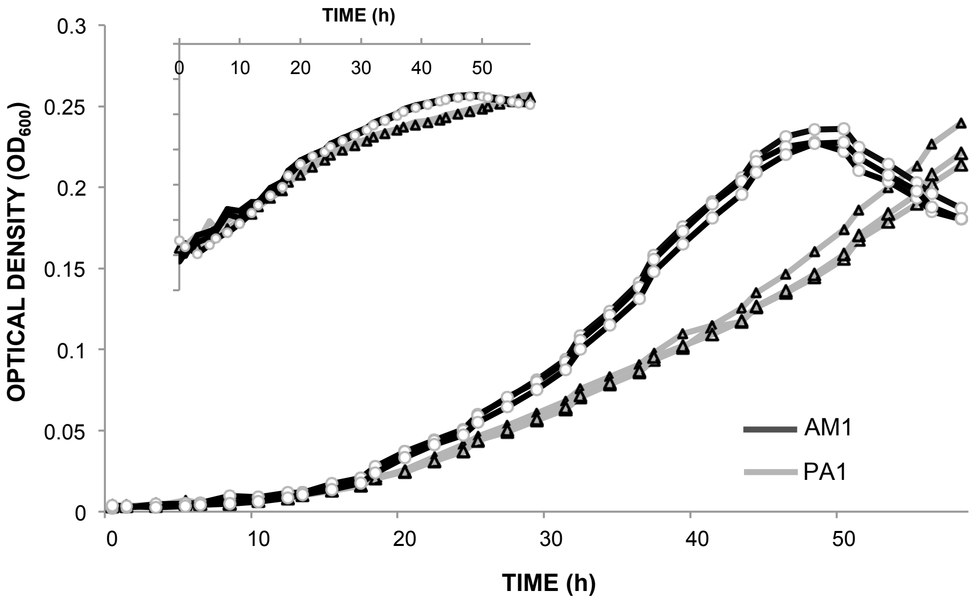

Supplement: Figure S2 — Growth of three biological replicates of the Δ cel ‘wild-type’ strain of PA1 (gray) and the Δ cel ‘wild-type’ strain of AM1 (black) in nutrient broth. The inset shows the semi-log plot of the growth curves to emphasize the deceleration in growth. (PDF) [file pone.0107887.s002.pdf]
