# Supplementary material for: Genetic and Phenotypic Comparison of Facultative Methylotrophy between Methylobacterium extorquens Strains PA1 and AM1
Source: PLoS One. 2014 Sep 18;9(9):e107887. doi: 10.1371/journal.pone.0107887 (PMC4169470; doi:10.1371/journal.pone.0107887)

Figure S3: Growth curves of three replicates of the Δ*cel* strain of PA1 (WT) (in green), the Δ*mxa* mutant of WT (in red), and the Δ*glyA* mutant of WT (in blue) on a combination of 7.5 mM methanol and 1.75 mM succinate as seen in the open-source growth curve fitter software, Curve Fitter.

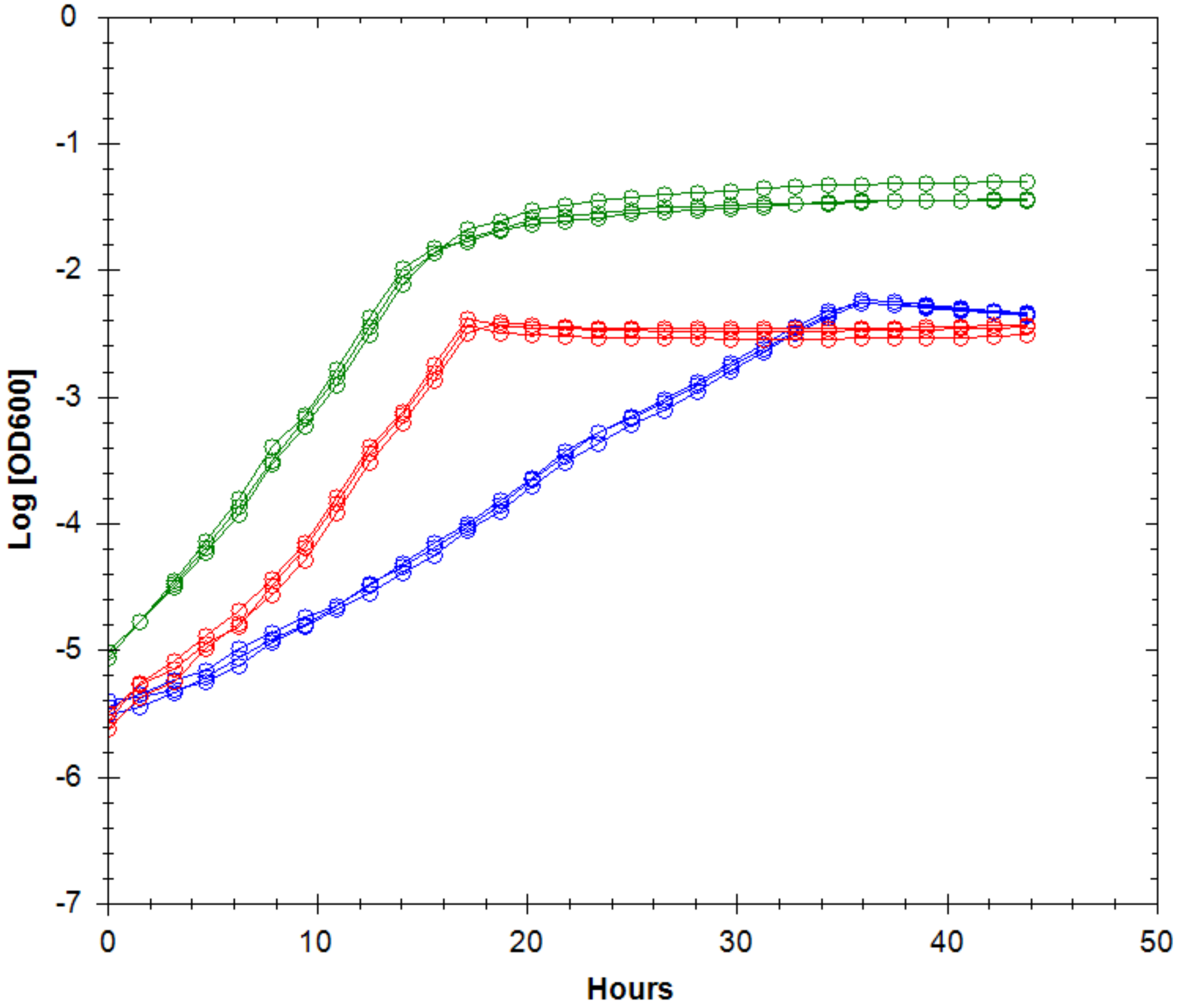

Supplement: Figure S3 — Growth curves of three replicates of the Δ cel strain of PA1 (WT) (in green), the Δ mxa mutant of WT (in red), and the Δ glyA mutant of WT (in blue) on a combination of 7.5 mM methanol and 1.75 mM succinate as seen in the open-source growth curve fitter software, Curve Fitter. (PDF) [file pone.0107887.s003.pdf]
