# Supplementary material for: Genetic and Phenotypic Comparison of Facultative Methylotrophy between Methylobacterium extorquens Strains PA1 and AM1
Source: PLoS One. 2014 Sep 18;9(9):e107887. doi: 10.1371/journal.pone.0107887 (PMC4169470; doi:10.1371/journal.pone.0107887)
Supplement: Table S1 — A list of methylotrophy-specific genes shared between M. extorquens strains AM1 and PA1. Green: genes involved in methanol oxidation, pink: genes involved in the H4MPT dependent formaldehyde oxidation pathway; purple: genes involved in the H4F-dependent formate reduction pathway; orange: genes encoding each of the four formate dehydrogenases; and gray: C1 assimilation genes. (PDF) [file pone.0107887.s004.pdf]

Table S1: A list of methylotrophy-specific genes shared between *M. extorquens* strains AM1 and PA1. Green: genes involved in methanol oxidation, pink: genes involved in the H_4MPT dependent formaldehyde oxidation pathway; purple: genes involved in the H_4F-dependent formate reduction pathway; orange: genes encoding each of the four formate dehydrogenases; and gray: C_1 assimilation genes.

Gene Name	Locus in AM1	Locus in PA1	% Amino Acid Identity
mxaB	**Meta1_4525**	**Mext_4137**	**100**
mxaH	**Meta1_4526**	**Mext_4138**	**98.46**
mxaE	**Meta1_4527**	**Mext_4139**	**96.82**
mxaD	**Meta1_4528**	**Mext_4140**	**99.43**
mxaL	**Meta1_4529**	**Mext_4141**	**97.59**
mxaK	**Meta1_4530**	**Mext_4142**	**98.56**
mxaC	**Meta1_4531**	**Mext_4143**	**99.15**
mxaA	**Meta1_4532**	**Mext_4144**	**96.42**
mxaS	**Meta1_4533**	**Mext_4145**	**96.89**
mxaR	**Meta1_4534**	**Mext_4146**	**99.42**
mxaI	**Meta1_4535**	**Mext_4147**	**100**
mxaG	**Meta1_4536**	**Mext_4148**	**99.49**
mxaJ	**Meta1_4537**	**Mext_4149**	**97.33**
mxaF	**Meta1_4538**	**Mext_4150**	**100**
mxaW	**Meta1_4539**	**Mext_4151**	**96.97**
mxbM	**Meta1_1752**	**Mext_1821**	**100**
mxbD	**Meta1_1753**	**Mext_1822**	**99.63**
mxcQ	**Meta1_4896**	**Mext_4452**	**99.19**
mxcE	**Meta1_4897**	**Mext_4453**	**100**
pqqE	**Meta1_1748**	**Mext_1817**	**99.47**
pqqCD	**Meta1_1749**	**Mext_1818**	**98.12**
pqqB	**Meta1_1750**	**Mext_1819**	**98.7**
pqqA	**Meta1_1751**	**Mext_1820**	**100**
dmrA	**Meta1_4312**	**Mext_3930**	**100**
mptG	**Meta1_1760**	**Mext_1828**	**99.4**
orfY	**Meta1_1762**	**Mext_1830**	**95.19**
orf5	**Meta1_1764**	**Mext_1832**	**99.67**
orf7	**Meta1_1765**	**Mext_1833**	**99.31**
orf17	**Meta1_1767**	**Mext_1835**	**98.16**
orf9	**Meta1_1768**	**Mext_1836**	**97.57**
orf19	**Meta1_1773**	**Mext_1841**	**97.47**
orf20	**Meta1_1774**	**Mext_1842**	**99.05**
orf21	**Meta1_1775**	**Mext_1843**	**95.1**
orf22	**Meta1_1776**	**Mext_1844**	**97.03**
fae	**Meta1_1766**	**Mext_1834**	**100**
mtdB	**Meta1_1761**	**Mext_1829**	**100**
fhcC	**Meta1_1755**	**Mext_1824**	**98.49**
fhcD	**Meta1_1756**	**Mext_1825**	**100**
fhcA	**Meta1_1757**	**Mext_1826**	**99.64**
fhcB	**Meta1_1758**	**Mext_1827**	**99.72**
mch	**Meta1_1763**	**Mext_1831**	**100**
drfA	**Meta1_2852**	**Mext_2659**	**96.99**

folK	Meta1_1743	Mext_1812	98.1
folB	Meta1_1744	Mext_1813	97.69
folP	Meta1_1745	Mext_1814	98.62
folC	Meta1_4888	Mext_4444	99.55
folE	Meta1_2264	Mext_2685	100
ftfL	Meta1_0329	Mext_0414	99.82
mtdA	Meta1_1728	Mext_1797	99.65
fch	Meta1_1729	Mext_1798	98.56
fdh3A	Meta1_0303	Mext_0389	99.9
fdh3B	Meta1_0304	Mext_0390	100
fdh3C	Meta1_0305	Mext_0391	99.42
fdh4B	Meta1_2093	Mext_2104	98
fdh4A	Meta1_2094	Mext_2105	98.96
fdh2C	Meta1_4846	Mext_4404	100
fdh2B	Meta1_4847	Mext_4405	98.84
fdh2A	Meta1_4848	Mext_4406	99.68
fdh1B	Meta1_5031	Mext_4581	99.65
fdh1A	Meta1_5032	Mext_4582	99.9
sga	Meta1_1726	Mext_1795	99.75
hprA	Meta1_1727	Mext_1796	99.68
ppc	Meta1_1732	Mext_1801	99.78
mtkA	Meta1_1730	Mext_1799	100
mtkB	Meta1_1731	Mext_1800	100
mcl	Meta1_1733	Mext_1802	100
glyA	Meta1_3384	Mext_3171	100
gck	Meta1_2944	Mext_2747	98.86
eno	Meta1_2984	Mext_2784	100
mdh	Meta1_1537	Mext_1643	100
qscR	Meta1_0756	Mext_0978	99.7
phaA	Meta1_3700	Mext_3469	100
phaB	Meta1_3701	Mext_3470	99.59
phaR	Meta1_3699	Mext_3468	99.01
croR	Meta1_3675	Mext_3444	100
pccA	Meta1_3203	Mext_2996	99.55
pccB	Meta1_0172	Mext_0282	99.8
ccr	Meta1_0178	Mext_0288	99.77
meaA	Meta1_0180	Mext_0290	99.56
meaB	Meta1_0188	Mext_0298	99.7
meaC	Meta1_4153	Mext_3781	99.14
meaD	Meta1_1432	Mext_1541	99.53
ibd2	Meta1_2223	Mext_2228	99.82
mcmA	Meta1_5251	Mext_4797	99.72
mcmB	Meta1_2390	Mext_2388	97.02
sdhA	Meta1_3861	Mext_3602	99.5
sdhB	Meta1_3863	Mext_3604	100
sdhC	Meta1_3859	Mext_3600	98.5
sdhD	Meta1_3860	Mext_3601	99.28
fumC	Meta1_1338	Mext_1449	99.81
