# Supplementary material for: Genetic and Phenotypic Comparison of Facultative Methylotrophy between Methylobacterium extorquens Strains PA1 and AM1
Source: PLoS One. 2014 Sep 18;9(9):e107887. doi: 10.1371/journal.pone.0107887 (PMC4169470; doi:10.1371/journal.pone.0107887)
Supplement: Table S2 — Mean FSC (Forward Scatter) and SSC (Side Scatter) of 50,000 cells of M. extorquens AM1 and PA1 (grown in 3.5 mM succinate) using a flow cytometer. FSC is an estimate for the relative size of the cell and SSC is an estimate for the granularity or the biomass/OD600 ratio of the cell. Values are reported as mean±95% confidence interval of the mean for three independent flow cytometer runs with 50,000 cells each. (PDF) [file pone.0107887.s005.pdf]

**Table S2:** Mean FSC (Forward Scatter) and SSC (Side Scatter) of 50,000 cells of *M. extorquens* AM1 and PA1 (grown in 3.5 mM succinate) using a flow cytometer. FSC is an estimate for the relative size of the cell and SSC is an estimate for the granularity or the biomass/$OD_{600}$ ratio of the cell. Values are reported as mean ± 95% confidence interval of the mean for three independent flow cytometer runs with 50,000 cells each.

| Strain | Mean FSC | Mean SSC |
|---|---|---|
| *M. extorquens* AM1 | 797±23 | 5923±219 |
| *M. extorquens* PA1 | 806±34 | 5422±321 |
