# Supplementary material for: Genetic and Phenotypic Comparison of Facultative Methylotrophy between Methylobacterium extorquens Strains PA1 and AM1
Source: PLoS One. 2014 Sep 18;9(9):e107887. doi: 10.1371/journal.pone.0107887 (PMC4169470; doi:10.1371/journal.pone.0107887)
Supplement: Table S4 — Mean growth rates (in h−1) and the standard srror of the mean growth rates on multi-C substrates S (3.5 mM succinate), P (5 mM pyruvate), E (7.5 mM ethanol) for AM1 and PA1 (both lacking the cel locus), as well as the mutants strains of Δ cel PA1. (PDF) [file pone.0107887.s007.pdf]

Table S4: Mean growth rates (in h^{-1}) and the standard srror of the mean growth rates on multi-C substrates S (3.5 mM succinate), P (5 mM pyruvate), E (7.5 mM ethanol) for AM1 and PA1 (both lacking the *cel* locus), as well as the mutants strains of Δ*cel* PA1.

Strains	S (h^{-1})	P (h^{-1})	E (h^{-1})
AM1	0.202±0.001	0.141±0.001	0
PA1	0.213±0.002	0.192±0.002	0.158±0.001
Δ*fae*	0.212±0.001	0.178±0.002	0.166±0.001
Δ*ftfL*	0.217±0.001	0.184±0.003	0.158±0.001
Δ*glyA*	0.194±0.002	0	0
Δ*mptG*	0.184±0.002	0.144±0.003	0.155±0.001
Δ*mxa*	0.213±0.002	0.185±0.001	0
Δ*hprA*	0.203±0.001	0.192±0.001	0.160±0.001
