# Supplementary material for: Genetic and Phenotypic Comparison of Facultative Methylotrophy between Methylobacterium extorquens Strains PA1 and AM1
Source: PLoS One. 2014 Sep 18;9(9):e107887. doi: 10.1371/journal.pone.0107887 (PMC4169470; doi:10.1371/journal.pone.0107887)
Supplement: Table S5 — Mean growth rates (in h−1) and the standard error of the mean growth rates on a joint C1 and multi-C substrate B (15 mM betaine), or a combination of C1 and multi-C substrates ½M+½S (7.5 mM methanol and 1.75 mM succinate) for AM1 and PA1 (both lacking the cel locus), as well as the mutants strains of Δ cel PA1. (PDF) [file pone.0107887.s008.pdf]

Table S5: Mean growth rates (in h^{-1}) and the standard error of the mean growth rates on a joint C_1 and multi-C substrate B (15 mM betaine), or a combination of C_1 and multi-C substrates ½ M + ½ S (7.5 mM methanol and 1.75 mM succinate) for AM1 and PA1 (both lacking the *cel* locus), as well as the mutants strains of Δ*cel* PA1.

Strains	B (h^{-1})	½ M + ½ S (h^{-1})
AM1	0.160±0.001	0.207±0.001
PA1	0	0.248±0.001
Δ*fae*	0	0
Δ*ftfL*	0	0.141±0.001
Δ*glyA*	0	0.125±0.001
Δ*mptG*	0	0
Δ*mxa*	0	0.236±0.002
Δ*hprA*	0	0.184±0.001
