# Supplementary material for: Genetic and Phenotypic Comparison of Facultative Methylotrophy between Methylobacterium extorquens Strains PA1 and AM1
Source: PLoS One. 2014 Sep 18;9(9):e107887. doi: 10.1371/journal.pone.0107887 (PMC4169470; doi:10.1371/journal.pone.0107887)
Supplement: Table S6 — Max OD600 and the standard error of the max OD600 on C1 substrates M (15 mM methanol), MA (15 mM methylamine), F (15 mM formate) for AM1 and PA1 (both lacking the cel locus), as well as the mutants strains of Δ cel PA1. (PDF) [file pone.0107887.s009.pdf]

Table S6: Max OD_{600} and the standard error of the max OD_{600} on C_1 substrates M (15 mM methanol), MA (15 mM methylamine), F (15 mM formate) for AM1 and PA1 (both lacking the *cel* locus), as well as the mutants strains of Δ*cel* PA1.

Strains	M (h^{-1})	MA (h^{-1})	F (h^{-1})
AM1	0.165±0.009	0.227±0.005	0.046±0.001
PA1	0.288±0.020	0.021±0.002	0.069±0.001
Δ*fae*	0	0	0.069±0.001
Δ*ftfL*	0	0	0
Δ*glyA*	0	0	0
Δ*mptG*	0	0	0.071±0.002
Δ*mxa*	0.012±0.001	0	0.067±0.001
Δ*hprA*	0	0	0
