# Supplementary material for: Genetic and Phenotypic Comparison of Facultative Methylotrophy between Methylobacterium extorquens Strains PA1 and AM1
Source: PLoS One. 2014 Sep 18;9(9):e107887. doi: 10.1371/journal.pone.0107887 (PMC4169470; doi:10.1371/journal.pone.0107887)
Supplement: Table S7 — Mean max OD600 and the standard error of the max OD600 on multi-C substrates S (3.5 mM succinate), P (5 mM pyruvate), E (7.5 mM ethanol) for AM1 and PA1 (both lacking the cel locus), as well as the mutants strains of Δ cel PA1. (PDF) [file pone.0107887.s010.pdf]

Table S7: Mean max OD_{600} and the standard error of the max OD_{600} on multi-C substrates S (3.5 mM succinate), P (5 mM pyruvate), E (7.5 mM ethanol) for AM1 and PA1 (both lacking the *cel* locus), as well as the mutants strains of Δ*cel* PA1.

Strains	S (h^{-1})	P (h^{-1})	E (h^{-1})
AM1	0.162±0.002	0.188±0.002	0.012±0.001
PA1	0.182±0.003	0.231±0.002	0.252±0.003
Δ*fae*	0.189±0.001	0.232±0.001	0.245±0.011
Δ*ftfL*	0.180±0.004	0.229±0.001	0.208±0.009
Δ*glyA*	0.178±0.006	0.048±0.001	0
Δ*mptG*	0.194±0.001	0.230±0.001	0.259±0.008
Δ*mxa*	0.177±0.006	0.226±0.002	0.022±0.002
Δ*hprA*	0.190±0.002	0.233±0.002	0.202±0.015
