# Supplementary material for: Genetic and Phenotypic Comparison of Facultative Methylotrophy between Methylobacterium extorquens Strains PA1 and AM1
Source: PLoS One. 2014 Sep 18;9(9):e107887. doi: 10.1371/journal.pone.0107887 (PMC4169470; doi:10.1371/journal.pone.0107887)
Supplement: Table S8 — Mean max OD600 and the standard error of the max OD600 on a joint C1 and multi-C substrate B (15 mM betaine), or a combination of C1 and multi-C substrates ½M+½S (7.5 mM methanol and 1.75 mM succinate) for AM1 and PA1 (both lacking the cel locus), as well as the mutants strains of Δ cel PA1. (PDF) [file pone.0107887.s011.pdf]

**Table S8:** Mean max $OD_{600}$ and the standard error of the max $OD_{600}$ on a joint $C_1$ and multi-C substrate B (15 mM betaine), or a combination of $C_1$ and multi-C substrates ½ M + ½ S (7.5 mM methanol and 1.75 mM succinate) for AM1 and PA1 (both lacking the *cel* locus), as well as the mutants strains of Δ*cel* PA1.

| Strains | B ($h^{-1}$) | ½ M + ½ S ($h^{-1}$) |
|---|---|---|
| AM1 | 0.384±0.013 | 0.155±0.006 |
| PA1 | 0 | 0.245±0.011 |
| Δ*fae* | 0 | 0 |
| Δ*ftfL* | 0 | 0.091±0.002 |
| Δ*glyA* | 0 | 0.103±0.001 |
| Δ*mptG* | 0 | 0 |
| Δ*mxa* | 0 | 0.087±0.002 |
| Δ*hprA* | 0 | 0.139±0.005 |
