# Supplementary material for: Genetic and Phenotypic Comparison of Facultative Methylotrophy between Methylobacterium extorquens Strains PA1 and AM1
Source: PLoS One. 2014 Sep 18;9(9):e107887. doi: 10.1371/journal.pone.0107887 (PMC4169470; doi:10.1371/journal.pone.0107887)
Supplement: Table S9 — List of primers used for deleting methylotrophy-specific genes in M. extorquens PA1. (PDF) [file pone.0107887.s012.pdf]

Table S9: List of primers used for deleting methylotrophy-specific genes in *M. extorquens* PA1.

Primer	Primer Sequence	Primer Description
Δ*fae*_us_f	ATGGATGCATATGCTGCAGCTCGAGCGGC CGCCGACGTGTCCCAAACCGATG	Gibson forward linker with Not1 and 24 bp of pCM433 backbone at 5' end. Product size = 695 bp
Δ*fae*_us_r	GTCCAAGGAAGATCGAAGCCAGTCGCTG GA GGTCTCTCCCTGGATTCCTG	30 bp of ds region linker at 5'end. Product size = 695 bp
Δ*fae*_ds_f	TCCAGCGACTGGCTTCGATC	Product size = 424 bp
Δ*fae*_ds_r	GGTTAACACGCGTACGTAGGGCCCGCGGC CGC CAGCGAGAGCCAGCTTGATG	Gibson reverse linker with Not1 and 24 bp of pCM433 backbone at 5' end. Product size = 424 bp
Δ*ftfL*_us_f	GTC CCT ATG CTT CCG TGG TAG C	Product size = 617 bp
Δ*ftfL*_us_r	GGTTAACACGCGTACGTAGGGCCCGCGGC CGC CGC AGA ACG TGT GGG TGA AAC G	Gibson reverse linker with Not1 and 24 bp of pCM 433 backbone. Product size = 617 bp
Δ*ftfL*_ds_f	ATGGATGCATATGCTGCAGCTCGAGCGGC CGCCGA CAT GAC GCT GCA TCT CTC C	30 bp of us region linker at 5'end. Product size = 451 bp
Δ*ftfL*_ds_r	TTCCGTTCGCTACCACGGAAGCATAGGGA CCCT GAC TTG GGC GGA TCG TTG	Gibson forward linker with Not1 and 24 bp of pCM433 backbone at 5' end. Product size = 451 bp
Δ*mptG*_us_f	ATGGATGCATATGCTGCAGCTCGAGCGGC CGCGAT CTC GGC GAT CAG CTC ACC	Gibson forward linker with Not1 and 24 bp of pCM433 backbone at 5' end. Product size =859 bp
Δ*mptG*_us_r	GCC GTT GAG ATC GAG GAA GCC	Product size = 859 bp
Δ*mptG*_ds_f	CTGCATTTCGGCTTCCTCGATCTCAACGGC CTC GCA GAA GGA GGC GGA	Gibson forward linker from 2049230 to 2049259 on PA1 genome. Product size = 723 bp
Δ*mptG*_ds_r	GGTTAACACGCGTACGTAGGGCCCGCGGC CGCGGT GAA GGC GAT CTT CGA GAC G	Gibson reverse linker with Not1 and 24 bp of pCM433 backbone at 5' end. Product size 723 bp
Δ*glyA*_us_f	GAT CAG CTC GAT CTC GTG CTG CTG C	Product size = 497 bp
Δ*glyA*_us_r	GGTTAACACGCGTACGTAGGGCCCGCGGC CGCTGA CGC TTC CGA TCG CAC GTG	Gibson Reverse Linker with Not1 and 24 bp of pCM433 backbone at 5'end. Product size = 497 bp
Δ*glyA*-ds_f	ATGGATGCATATGCTGCAGCTCGAGCGGC CGCCGC GCA ACC TTC AGG TGA AGG ATG G	Gibson forward linker with Not1 and 24 bp of pCM 433 backbone at 5'end. Product size = 526 bp
Δ*glyA*_ds_r	GGT ACC GAC AAC CAC CTG ATG CTG G	Product size = 526 bp
Δ*mxa*_us_f	ATGGATGCATATGCTGCAGCTCGAGCGGC CGCGAT CGA GGT GCA ACT CGG CAG	Gibson forward linker with Not1 and 24 bp of pCM433 backbone at 5' end. Product size = 701bp
Δ*mxa*_us_r	TGG TCC AGA TCG CGG TCA AC	Product size = 701bp
Δ*mxa*_ds_f	CGTTGCGCCGGTTGACCGCGATCTGGACC ATCG TGA TGC TGA ACG CGC AC	Gibson linker with 4606355 through 4606384 on PA1 genome. Product size = 496bp
Δ*mxa*_ds_r	GGTTAACACGCGTACGTAGGGCCCGCGGC CGCAAC TCG ATG TCG AAG GCG TGC	Gibson reverse linker with Not1 and 24 bp of pCM433 backbone at 5' end. Product size = 496bp
Δ*hprA*_us_f	ATGGATGCATATGCTGCAGCTCGAGCGGC CGCCTC ATC GAC AAC GGC GTG AAG G	Gibson forward linker with Not1 and 24 bp of pCM433 backbone at 5' end. Product size = 344bp
Δ*hprA*_us_r	GGGCAATCGTGTCGCTCAC	Product size = 344bp
Δ*hprA*_ds_f	GCAGGGGTTTTGTGAGCGACACGATTGCC CCTCGTGGACAACGTCGAAGC	30 bp from 2016078 to 2016107 on PA1 genome at 5' end. Product size = 447bp
Δ*hprA*_ds_r	GGTTAACACGCGTACGTAGGGCCCGCGGC CGCTCT TCA CCG CCT CGA ACA CC	Gibson reverse linker with Not1 and 24 bp of pCM433 backbone at 5' end. Product size = 447bp
Δ*fhc*_us_f	ATGGATGCATATGCTGCAGCTCGAGCGGC CGCCCG AGA TGC TTG AGG CTC TG	Gibson forward linker with Not1 and 24 bp of pCM433 backbone at 5' end. Product size = 480bp
Δ*fhc*_us_r	GCT CTC GCA AGG CTC GAT CC	Product size = 480bp
Δ*fhc*_ds_f	CCCTCGACCCGGATCGAGCCTTGCGAGAG CTCC GGG TCG TAG ACG ATG AC	30 bp from 2044769 to 2044798 on PA1 genome at 5' end. Product size = 569bp
Δ*fhc*_ds_r	GGTTAACACGCGTACGTAGGGCCCGCGGC CGCTGA GGA AGC ATG GCA GCC TG	Gibson reverse linker with Not1 and 24 bp of pCM433 backbone at 5' end. Product size =569bp
